# Mesenteric Ischemia: A Comprehensive Review of the Role of Radiology in Diagnosis

**DOI:** 10.7759/cureus.90384

**Published:** 2025-08-18

**Authors:** Samuel I. Dos Santos Pereira, Amnah Alshatti, Nourhane Al Akoum, Seeme Rukh, Noor Q Malik, Saqib Ahmed, Rukhsimran Kaur, Humza F Siddiqui

**Affiliations:** 1 Department of Internal Medicine, University of Buenos Aires, Buenos Aires, ARG; 2 General Surgery, Adan Hospital, Al Ahmadi, KWT; 3 Medicine, Beirut Arab Univeristy, Beirut, LBN; 4 Medicine, Malik Haider Hospital, Gujrat, PAK; 5 Internal Medicine, Maimonides Medical Center, New York City, USA; 6 Internal Medicine, Allama Iqbal Medical College, Gujrat, PAK; 7 Medicine and Surgery, SDM College of Medical Sciences and Hospital, Dharwad, IND; 8 Medicine and Surgery, Teerthanker Mahaveer University, Moradabad, IND; 9 Internal Medicine, Jinnah Postgraduate Medical Centre, Karachi, PAK

**Keywords:** acute mesenteric ischemia (ami), chronic mesenteric ischemia (cmi), ct angio, duplex ultrasound, inferior mesenteric artery, magnetic resonance imaging, non-occlusive mesenteric ischemia, superior mesenteric artery

## Abstract

Acute mesenteric ischemia (AMI) is an uncommon yet immensely fatal cause of abdominal pain with significant clinical consequences. Chronic mesenteric ischemia (CMI) accounts for a small proportion of intestinal ischemic cases but has significant clinical consequences. The majority of AMI occurs due to occlusive etiologies, including arterial and venous emboli and thrombus. Non-occlusive causes resulting in decreased blood supply to the intestines constitute the remaining cases. CMI results from an indolent formation of atherosclerotic plaques in the superior and inferior mesenteric arteries, pertaining to risk factors including hypertension, hyperlipidemia, and advanced age. Timely diagnosis of mesenteric ischemia is crucial and can aid in curtailing the associated morbidity and mortality. This review aims to provide an overview of the role of radiological modalities in diagnosing AMI and CMI. Computed tomography (CT) is the most widely accepted modality utilized to detect intestinal ischemia in clinically suspected cases. CT angiography (CTA) has shown the best results in identifying the vascular findings, delineating the primary etiologies. Additionally, several non-vascular CT findings have also been reported. Bowel wall thickening, mesenteric stranding, and ascites are common but non-specific findings that correlate poorly with disease severity. Pneumatosis intestinalis and porto-mesenteric venous gas, while not pathognomonic for ischemia, are highly specific in cases of high clinical suspicion. Exposure to radiation, nephropathy, and allergic reactions to contrast agents limit the use of CT in certain cases. Duplex ultrasound and magnetic resonance imaging have proved to be useful among these patients. However, each imaging modality has its limitations and challenges that need to be addressed. It is imperative to formulate definitive guidelines regarding the use of radiological tools compounded with other clinical and laboratory markers to help clinicians timely diagnose and treat mesenteric ischemia to enhance patient outcomes.

## Introduction and background

Acute mesenteric ischemia (AMI) and chronic mesenteric ischemia (CMI) are less prevalent gastrointestinal (GIT) conditions globally but have high morbidity and mortality rates [1]. The prevalence of AMI is 0.1% and the incidence ranges between 5.3 and 8.4 cases per 100,000 per year. CMI accounts for less than 1 in 1000 hospital admissions related to abdominal pain [2]. A study exhibited that five out of every 10,000 hospital admissions were due to AMI [3]. Despite being uncommon, these conditions have an exponentially increasing incidence with age and are more prevalent among the female population [4]. However, recent advancements in imaging and healthcare training are leading to early diagnosis and management of AMI, reducing mortality significantly [1].

AMI, a fatal vascular emergency, is the result of the interruption of blood flow in the superior mesenteric artery, which supplies most of the gastrointestinal tract. (3). Untreated ischemia leads to life-threatening necrosis with a 50% mortality rate [5]. AMI should be suspected when a patient presents with a sudden onset of severe abdominal pain out of proportion to physical findings. CMI, also known as abdominal angina, is mainly a disease of the elderly population attributable to higher cardiovascular events and risk factors [4]. Postprandial angina is the most frequent typical symptom of CMI. Patients develop sitophobia with altered eating behaviors leading to weight loss [5]. AMI and CMI have a substantial in-hospital mortality of 63%. Therefore, timely diagnosis of mesenteric ischemia prior to the development of bowel infarction is crucial for favorable patient outcomes [4]. Diagnosis of AMI and CMI is challenging due to the wide array of differentials, with a high index of suspicion required to timely diagnose the condition. An interdisciplinary team is needed to devise an optimal management strategy [5]. The gold standard for diagnosis is computed tomography angiography (CTA) with a substantially high sensitivity and specificity [4, 6]. CTA aids in providing an accurate, timely diagnosis and also delineates the accountable underlying etiology [4]. It reveals an obstructing embolus or atherosclerotic plaque in the artery, and thickening and dilation in the intestinal wall [1, 6, 7].

Timely diagnosis is crucial to prevent bowel infarction, as it leads to invasive surgical bowel resection, resulting in a 50% post-resection five-year survival rate [4]. Nonspecific symptoms and the wide differential diagnosis for abdominal pain, in parallel with limited expertise among healthcare providers, make diagnosis challenging. The delay in diagnosis reflects a delay in treatment, thus impacting the prognosis of patients negatively [8]. This is achievable by formulating diagnostic guidelines that will aid healthcare professionals in providing prompt diagnosis, preventing disease progression. A patient-centered approach is the mainstay of having promising survival outcomes. Controlling patient risk factors has been shown to reduce death by 40% among CMI patients [9]. This review aims to delineate the findings and performance of radiological modalities to provide an overview of the diagnostic role of these modalities in AMI and CMI.

## Review

Anatomy of the blood supply

The celiac axis, superior mesenteric, and inferior mesenteric vessels form the splanchnic vasculature [10]. The common hepatic, splenic, and left gastric arteries that come off the celiac axis supply the stomach, pancreas, and spleen. The common hepatic artery gives rise to the gastroduodenal artery [11]. The duodenum distal to the duodenal papilla, the rest of the small intestine, and the large intestine up to the anterior two-thirds of the transverse colon receive the blood distribution from branches of the superior mesenteric artery (SMA) [5]. The remaining transverse colon, left colon, and two-thirds of the rectum receive blood from the inferior mesenteric artery (IMA) [12]. There is a vast collateral circulation among the three main branches to ensure adequate blood flow. The gastroduodenal and pancreaticoduodenal arteries (PDA) connect the celiac axis and the SMA. The middle and left colic arteries originate from the SMA and IMA, respectively. These two branches then form an anastomosis to supply the splenic flexure, a watershed region, also called Griffith Point. The arc of Riolan and the marginal arcade are also the point of anastomosis of the SMA and IMA. The marginal arcade is formed from a conjunction of the ileocolic, right middle, and left colic arteries. The central anastomotic artery is formed by the conjunction of the middle and left colic arteries. Further down, the superior, middle, and inferior rectal arteries form the Sudeck's point, which supplies blood to the sigmoid colon. The superior rectal artery is a branch of the IMA, while the middle and inferior rectal arteries stem off of internal iliac arteries [6]. The most prone areas to ischemia include the ascending colon, Griffith point, and Sudeck's point [13]. The venous drainage system is adjacent to the arterial system, consisting of the superior and inferior mesenteric veins. The ileum, jejunum, right and transverse colon are drained by the superior mesenteric vein (SMV). The left and rectosigmoid colon are drained by the inferior mesenteric vein (IMV). The SMV and the splenic vein merge to form the portal vein. IMV can either fall into SMV, splenic vein, or their conjunction [14]. The portal vein enters the liver, the blood then flows through the hepatic sinusoids and is eventually drained via hepatic veins into the inferior vena cava. The splanchnic vasculature receives 15 to 30% of the cardiac output. The blood flow has to be decreased to more than 50% for the intestinal ischemia to develop [15].

Etiology of mesenteric ischemia

Arterial embolism accounts for 40-50% of the cases of AMI. SMA is the most commonly occluded and is responsible for about 5% of emboli. Atrial fibrillation (AF) and myocardial infarction (MI) commonly precede embolic intestinal ischemia due to the dislodging of left atrial thrombus and mural thrombus, respectively. Thrombus detachment from the valve in endocarditis is another cause. It is usually associated with complete obstruction, with the onset of necrosis within six hours of onset, as collateral circulation is absent [16]. Arterial Thrombus contributes to 25-30% of AMI cases. The implicated pathology is thrombosis occurring atop existing atherosclerotic plaques in the mesenteric arteries, especially the SMA. Development of collateral circulation delays the manifestation of symptoms until stenosis reaches >70%. Progressive atherosclerotic narrowing reaches a critical threshold where acute events predisposing to thrombosis accelerate the occurrence of ischemia. Risk factors include hypertension, hyperlipidemia, diabetes, smoking, and advanced age [17]. Non-occlusive mesenteric ischemia (NOMI) is responsible for the remaining 20-30% of AMI cases. Vasoconstriction of the splanchnic arterial bed secondary to low-output cardiovascular states and/or the use of vasopressors is usually the common offending etiology. It is usually associated with critically ill patients. Mesenteric venous thrombosis is a rare cause of intestinal ischemia. Thrombosis of mesenteric veins leads to venous obstruction and congestion, leading to ischemia. Conditions predisposing are hypercoagulable states, infections, and malignancy [4]. Development of porto-splenic mesenteric venous thrombosis (PSMVT) has been associated with COVID-19-infected patients. Risk factors in these patients include pre-existing comorbidities, such as advanced age, diabetes mellitus, cardiac disease, and hypertension [18].

**Figure 1:**
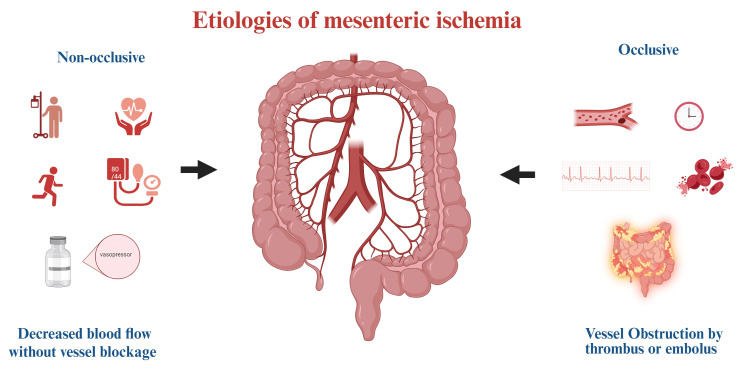
Etiologies of mesenteric ischemia Image credit: This figure was made by author Samuel Pereira using biorender.com

Chronic mesenteric ischemia (CMI) results from a gradual reduction in blood flow to the bowel, typically due to stenosis and obstruction by atherosclerotic disease. Other causes include median arcuate ligament syndrome (MALS) [3]. Progressive narrowing of the mesenteric arteries due to atherosclerotic plaque buildup leads to reduced perfusion, especially postprandially when intestinal blood demand increases. Symptoms typically emerge after two to five years of significant stenosis (>70%) in multiple vessels [19]. Smoking, hyperlipidemia, hypertension, and advanced age are significant high-risk contributors [3]. In chronic mesenteric NOMI (CNOMI), inadequate splanchnic circulation has been implied as the most probable cause, displaying symptoms due to mucosal ischemia without the presence of obvious vessel occlusion [4]. Figure 1 shows the etiologies of mesenteric ischemia.

Imaging modalities

Computed Tomography and Computed Tomography Angiography

Computed tomography (CT) is the most widely used imaging modality in clinically suspected acute mesenteric ischemia. However, in critically ill patients, the sensitivity of these imaging findings may be reduced, underscoring the need for continued clinical vigilance. A systematic review including 1970 patients demonstrated a pooled sensitivity of 94% and specificity of 95% in detecting AMI [20]. Similarly, another systematic review assessing the performance of multi-slice spiral CT in diagnosing AMI showed a pool sensitivity of 94%, specificity of 97%, and the area under the curve (AUC) of 0.99 [21]. The specificity of intestinal pneumatosis has been reported to reach approximately 99%, with a corresponding sensitivity of only 15%. For porto-venous gas, the specificity is estimated at 96%, and the sensitivity at 23%. Abnormal contrast enhancement of the bowel wall is also recognized as a significant CT indicator, with a sensitivity of 21% and specificity of 89% [22]. A recent study showed that multi-slice CT detected AMI 1.5 times more accurately than CMI, while occlusive and non-occlusive mesenteric ischemia was detected equally. CT also correctly identified celiac artery compression and chronic ischemic enterocolitis as the cause of CMI among 16% and 23% of the patients, respectively [23]. Multi-detector computed tomography angiography (MDCTA) is considered the first-line imaging modality in patients with suspected AMI. It is capable of accurately detecting arterial and venous thrombosis, assessing the extent of gastrointestinal involvement, and providing detailed information regarding the subtype and progression of the disease [24]. MDCTA has been shown to exhibit a sensitivity of 89.4% and a specificity of 99.5% in diagnosing AMI. CTA diagnosed 60% of the mesenteric ischemia cases as compared to 20% diagnosed clinically [25]. A systematic review revealed that CTA has the most superior performance in accurately diagnosing AMI as compared to other CT protocols, with a sensitivity of 92% and a specificity of 98.8% [26]

Vascular findings: The small takeoff angle of the SMA is highly susceptible to emboli compared to the celiac artery or IMA. Arterial emboli and thrombi appear as low attenuation filling defects in the affected artery [27]. A low attenuation filling defect within the Porto mesenteric venous system is indicative of thrombosis in the portal or splenic veins [27, 28], along with an engorgement of the collateral mesenteric venous pathways [10]. In cases of NOMI, the arteries remain patent with no intraluminal filling defect. However, vasoconstriction is observed in the splanchnic arteries, which is most of the time reversible, and the vessel caliber returns to its original diameter when the cause is treated [28]. An additional radiological finding is solid organ infarction, which helps include NOMI as a differential diagnosis of acute abdominal pain [29]. A study revealed that fewer than five branches of SMA and decreased venous return in two zones visible on CTA are prognostic indicators of mortality [30].

Bowel wall findings: In arterial ischemia, the bowel wall may appear paper-thin due to the loss of tissue, vessels, and muscle tone. Another feature is a decrease or partial uptake of contrast, which indicates impaired perfusion signifying bowel ischemia [9]. In cases of reperfusion, bowel wall thickening indicates revascularization. Pneumatosis intestinalis (PI), the presence of air within the bowel wall, occurs as a result of mucosal breakdown preceding irreversible necrosis. If the air appears as intramural cystic-like gas collections, this is considered benign pneumatosis, which may require no intervention [27]. However, a band-like distribution of PI is associated with transmural infarction and may indicate irreversible necrosis, especially when associated with portomesenteric venous gas [6, 31]. CT identified bowel wall thinning and porto-mesenteric venous gas with a specificity of 98% and 95% respectively, among patients with AMI [7].

In venous ischemia, bowel wall thickening and hyper-enhancement are often seen. Venous obstruction with maintained arterial perfusion causes vascular congestion, submucosal swelling, and tissue cyanosis. This creates a characteristic trilaminar pattern, where the hypo-enhancing submucosa is positioned between the hyper-enhancing mucosa and muscularis propria/serosa, commonly known as the "target sign" [6, 31]. In NOMI, coexistence of necrosis and perfusion can occur, which gives numerous CT findings alternating from bowel wall thickening and thinning and increased or decreased bowel enhancement. Pneumatosis is also frequently found in NOMI [17].

In cases of closed-loop obstruction, frequently resulting from adhesions or abdominal hernias, multiple dilated U- or C-shaped, fluid-filled bowel loops may be identified. When volvulus occurs, the twisting of the mesentery produces the characteristic "whirlpool sign", while intussusception may present with the distinctive "target sign" [32]. Bowel mural hypo-enhancement and the presence of mural hemorrhage are considered specific findings of strangulation obstruction [10].

Mesenteric findings: Regional mesenteric fat inflammation and fluid accumulation between the mesenteric layers strongly indicate transmural infarction [31]. Edema and congestion of the mesenteric fat would be visualized as haziness on CT in cases of venous-caused ischemia. Occasional fat stranding or ascites may be seen depending on the predisposing factors in NOMI [28]. Table 1 shows computed tomography findings in mesenteric ischemia.

**Table 1:** Computed tomography findings in mesenteric ischemia. The authors created the table using references [27-32].

NOMI	Venous	Arterial	
Vasoconstriction in the splanchnic arteries. No intraluminal filling defect identified.	Low attenuation intraluminal filling defect in porto-mesenteric venous system and collateral engorgement	Low attenuation intraluminal filling defect in the affected artery	Vessel
Segmental areas of reduced enhancement, dilated bowel loops, and wall thickening with mucosal hyperenhancement following vascular reperfusion or restoration	Thickening of the wall and hyper enhancement. Target sign can be identified	Thinning with diminished enhancement the wall. Presence of pneumatosis intestinalis	Bowel
Occasional fat stranding and ascites	Haziness due to venous congestion	Focal mesenteric fat stranding with ascites	Mesentery

CT protocols: Unenhanced CT scans are an essential component of the protocol and should precede contrast-enhanced studies. Their primary role is to identify intramural calcifications, intravascular thrombi, and signs of intramural hemorrhage. However, they should not delay the administration of contrast-enhanced scans, as such delays may increase mortality risk [33, 34].

A biphasic contrast-enhanced CT protocol is typically employed. It consists of two phases. An arterial phase acquired approximately 30 seconds post-injection, and a portal venous phase acquired at 60-70 seconds post-injection. High-flow intravenous injection of nonionic iodinated contrast (4-5 mL/s), followed by a saline flush (1.5 mL/kg), is utilized. Bolus tracking is employed to optimize timing. This biphasic approach allows for rapid and accurate detection of vascular filling defects, assessment of mesenteric arterial and venous structures, and evaluation of bowel wall enhancement [24, 34].

Duplex Ultrasound

Duplex ultrasound (DUS) has high specificity (92-100%) but lower sensitivity (70-80%) for detecting vascular occlusions, particularly non-occlusive thrombi and distal occlusions [35]. In advanced cases, findings such as intramural or portal vein gas may be less evident. While DUS can help narrow the causes of abdominal pain, its time-consuming nature and high failure rate make it unreliable for diagnosing AMI [36]. DUS is primarily used for diagnosing CMI, with peak systolic flow velocities above 295 cm/s in the celiac artery and above 240 cm/s in the SMA indicating hemodynamically significant (>50%) stenosis [17, 37]. One small study reported that detecting normal flow velocities in the celiac artery and SMA using bedside US may help exclude AMI, particularly in occlusive cases [38]. Contrast-enhanced ultrasound (CEUS) for mesenteric artery evaluation remains an off-label application in the United States [39]. CEUS demonstrated that partial lack of bowel wall enhancement was a sensitive imaging feature among surgically confirmed NOMI patients [34].

Despite these potential applications, US is highly operator-dependent and may be unreliable in visualizing mesenteric arteries due to factors such as bowel gas shadowing, dense vascular calcifications, and patient body habitus [34]. Ultrasound is a helpful tool for early detection of changes in the colonic wall due to ischemia. It can support diagnosis when used in the right clinical setting. It allows visualization of the location and length of the affected bowel segment and can show signs such as wall thickening, layering, and abnormal fat and fluid around the bowel. Using color Doppler, ultrasound can help distinguish between thickening caused by inflammation versus ischemia, and may even help identify patients at risk of necrosis [40]. However, ultrasound may miss early signs of ischemia. Also, features like pneumatosis intestinalis, which are significant on CT, are rarely seen on ultrasound [41].

A longitudinal multicentric cohort study demonstrated that the SMA peak systolic velocity (PSV) on DUS yielded a sensitivity of 78.6% and a specificity of 64.5% for the diagnosis of AMI. Notably, in cases of occlusive AMI, DUS achieved a sensitivity of 100% and a specificity of 64% [38]. Additionally, a study assessing DUS criteria for detecting in-stent restenosis (ISR) of the SMA concluded that a PSV threshold of 400 cm/sec on mesenteric DUS is predictive of significant ISR with high specificity and should be considered a reliable diagnostic criterion [42].

Magnetic resonance imaging and magnetic resonance angiography:

In cases of chronic mesenteric ischemia (CMI), invaluable substitutions include magnetic resonance imaging (MRI) as well as magnetic resonance angiography (MRA). Utilizing MRI exhibits more than 95% sensitivity and specificity for CMI in addition to furnishing insight into postprandial mesenteric blood flow. A 2D phase-contrast MRI, utilized in earlier studies, helped to differentiate healthy subjects from CMI patients based on changes in mesenteric arterial and venous flow following a meal challenge [10]. Functional and anatomical imaging, combined through the use of gadolinium-enhanced MRA, has its limitations, which are inclusive of longer acquisition times, heightened difficulty imaging through calcification or stents, as well as the risk of nephrogenic systemic fibrosis in those individuals diagnosed with renal dysfunction [43, 44]. While CTA and MRA both provide comparable sensitivity, stenosis severity can be overestimated due to their lower spatial resolution, in addition to limited scanner accessibility. Vascular specialists are more cognizant of CT and its vast availability, which promotes it as a dominant tool in conventional practice [44].

While CT remains the modality of choice for evaluating AMI, MRI offers a valuable alternative in select cases, particularly when CT is contraindicated due to allergy to iodinated contrast or concerns regarding ionizing radiation. Although MRI protocols for bowel ischemia are less standardized, specific sequences have shown diagnostic utility. T1-weighted sequence (T1WI) is useful in detecting hemorrhage and fat infiltration. T2-weighted sequence (T2WI) is effective in evaluating bowel wall edema and intra-abdominal fluid collections. Diffusion-weighted imaging (DWI) helps identify ischemic segments through the detection of restricted diffusion. Contrast-enhanced MR angiography (CE-MRA) enables visualization of mesenteric vessels and assessment for occlusion or stenosis. MRI remains a complementary modality in the diagnostic workup of bowel ischemia, particularly in chronic cases or when functional vascular evaluation is required [34].

Challenges and limitations

**Figure 2:**
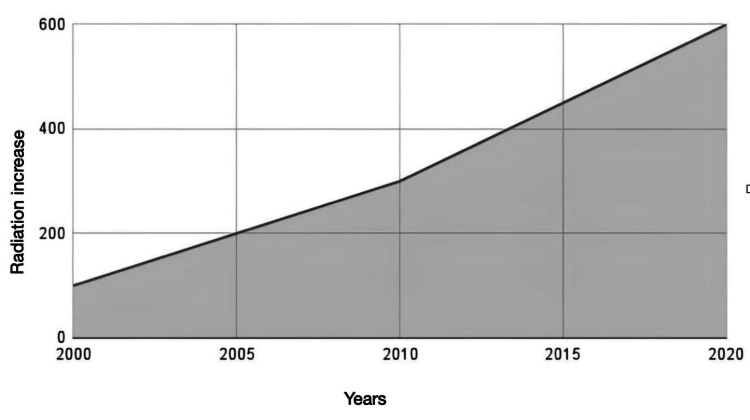
Evolution of radiation increase over time Image credit: This figure was created by authors Samuel Pereira and Seeme Rukh using Google Sheets (Google, Mountain View, California), inspired by reference [45].

Radiological interventions are essential in diagnosing and treating vascular and ischemic intestinal syndromes. However, certain challenges are faced, including technological limitations and imaging constraints, diagnostic uncertainty, patient-related factors, and logistical and systemic issues. One of the technological limitations of diagnostic tools is the exposure of patients with mesenteric ischemia to radiation from diagnostic methods such as CT scans. In the last two decades, with the innovation of novel imaging techniques, predisposition to radiation has escalated as much as 600 percent. In the first 10 years of life, children are more affected by exposure to radiation due to their sensitive tissues. This challenge can be tackled by introducing newer techniques, such as lower-dose CT scans or replacing them with techniques involving no radiation exposure, such as certain US and MRI modalities. Figure 2 demonstrates that radiation exposure over the last two decades has followed a consistent upward trend [45].

**Figure 3:**
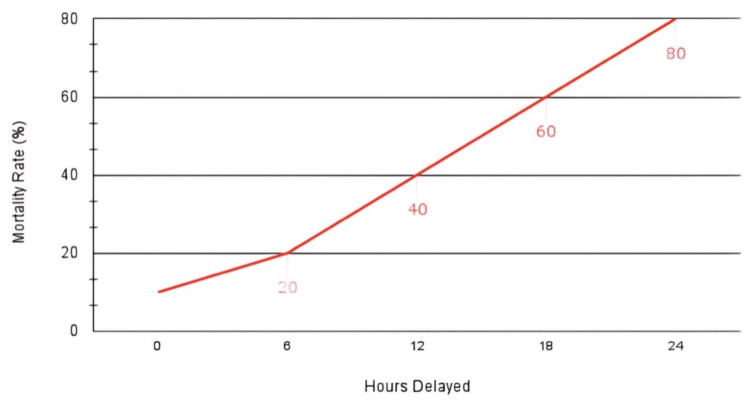
Mortality rate increase with delayed diagnosis of mesenteric ischemia Image credit: This figure was created by authors Samuel Pereira and Seeme Rukh using Google Sheets (Google, Mountain View, California), inspired by reference [15].

Radiation exposure is one of the challenges encountered when diagnosing mesenteric ischemia. Newer scans, such as split-bolus CTA, have been introduced to reduce radiation exposure. According to a retrospective study done in Europe, split-bolus CTA reduces radiation exposure while having a sensitivity of 100 percent and specificity of 99 percent for detecting mesenteric ischemia [46]. The split-bolus method is becoming increasingly common. It includes visualization of the venous and arterial phases simultaneously. It reduces exposure to the contrast material, and a diagnosis can be made with a couple of images [10]. Certain diagnostic techniques have limitations. Radiography, for example, is not the preferred mode of imaging for mesenteric ischemia as initial imaging may show vague changes such as the absence of gas in the abdomen to diffuse expansion as seen in the ileus [34]. X-ray and abdominal ultrasound are considered the top choice tests in cases of acute-onset abdominal pain, but their results cannot yield a diagnosis in many cases of mesenteric ischemia [14]. One of the challenges in diagnosing and treating intestinal ischemia on time is the lack of advanced and expensive imaging options in resource-limited settings. Delayed diagnosis, notably every passing six hours, increases the mortality rate two times, as demonstrated in Figure 3 [15]. 

Computed tomography angiography is known to cause contrast-induced nephropathy. However, the underlying mechanism needs further studies to be understood completely. Healthcare providers should always be aware of at-risk patient populations and ensure that such patients are hydrated before and after the procedure. The use of sodium bicarbonate and N-acetylcysteine to avoid contrast-induced nephropathy is controversial [47]. As mesenteric ischemia has high mortality and morbidity if it is not diagnosed and treated promptly, the benefits of appropriate diagnosis using contrast are more than the harm that might be caused to the kidney by contrast media [48]. Intrusive techniques such as catheter angiography are restricted solely to catheter-mediated thrombolysis if infarction is not present. With the evolution in CT scan techniques, catheter angiography is used for treatment and no longer for diagnostic purposes, as less invasive procedures are preferred [10].

Transabdominal DUS is not very accurate, as imaging of mesenteric vessels can be quite arduous in certain patients because of variations in anatomy and the presence of comorbidities. Even in cases of less than 20% occlusion of the superior mesenteric artery, consistent elevation of peak systolic velocity up to 335 cm/sec can be seen [49]. Even with the best imaging techniques, many abdominal diseases show overlapping features on imaging, and intestinal ischemia is either missed or misdiagnosed as another condition. Bowel ischemia, notably in the form of mesenteric vessel hyperplasia, often on imaging appears as inflammation of the small bowel or colon, thus adequate diagnosis is not made, and results can be detrimental [50]. Conditions such as ulcerative colitis, bacterial infections, enterocolitis, and bowel edema also sometimes look similar to acute intestinal ischemia on CT angiography [51].

## Conclusions

AMI and CMI are relatively uncommon causes of abdominal pain but have significant clinical consequences. Early and accurate detection of intestinal ischemia is crucial in the clinical setting to improve favorable patient outcomes. Radiological evaluation stands as a diagnostic tool utilized for the identification of intestinal ischemia. CT and CTA are the most widely accepted radiological modalities. US and MRI also show potential in identifying relevant findings. However, each imaging modality has its limitations and challenges that needs to be addressed. It is imperative to formulate definitive guidelines regarding the use of radiological tools compounded with other clinical and laboratory markers to help clinicians timely diagnose and treat mesenteric ischemia to enhance patient outcomes.
